# Supplementary material for: Associations between WASH-related violence and depressive symptoms in adolescent girls and young women in South Africa (HPTN 068): a cross-sectional analysis
Source: BMJ Open. 2022 Jul 5;12(7):e061032. doi: 10.1136/bmjopen-2022-061032 (PMC9258484; doi:10.1136/bmjopen-2022-061032)
Supplement: Author's manuscript [file bmjopen-2022-061032.draft_revisions.pdf]

BMJ Open is committed to open peer review. As part of this commitment we make the peer review history of every article we publish publicly available.

When an article is published we post the peer reviewers' comments and the authors' responses online. We also post the versions of the paper that were used during peer review. These are the versions that the peer review comments apply to.

The versions of the paper that follow are the versions that were submitted during the peer review process. They are not the versions of record or the final published versions. They should not be cited or distributed as the published version of this manuscript.

BMJ Open is an open access journal and the full, final, typeset and author-corrected version of record of the manuscript is available on our site with no access controls, subscription charges or pay-per-view fees (http://bmjopen.bmj.com).

If you have any questions on BMJ Open's open peer review process please email
info.bmjopen@bmj.com

BMJ Open

WASH-related violence and depressive symptoms in adolescent girls and young women in HIV prevention trials network 068

Journal:	*BMJ Open*
Manuscript ID	bmjopen-2022-061032
Article Type:	Original research
Date Submitted by the Author:	15-Jan-2022

Complete List of Authors:	Jayaweera, Ruvani T.; Ibis Reproductive Health<br>Goin, Dana; University of California San Francisco, Obstetrics, Gynecology, & Reproductive Sciences<br>Twine, Rhian; University of the Witwatersrand,<br>Neilands, Torsten; University of California San Francisco<br>Wagner, Ryan; University of the Witwatersrand<br>Lippman, Sheri ; University of California San Francisco<br>Kahn, Kathleen; University of the Witwatersrand,<br>Pettifor, Audrey; University of North Carolina at Chapel Hill Gillings School of Global Public Health, Epidemiology<br>Ahern, Jennifer; University of California Berkeley School of Public Health, Division of Epidemiology

Keywords:	EPIDEMIOLOGY, MENTAL HEALTH, Depression & mood disorders < PSYCHIATRY, PUBLIC HEALTH, Community child health < PAEDIATRICS

SCHOLARONE™
Manuscripts

I, the Submitting Author has the right to grant and does grant on behalf of all authors of the Work (as defined in the below author licence), an exclusive licence and/or a non-exclusive licence for contributions from authors who are: i) UK Crown employees; ii) where BMJ has agreed a CC-BY licence shall apply, and/or iii) in accordance with the terms applicable for US Federal Government officers or employees acting as part of their official duties; on a worldwide, perpetual, irrevocable, royalty-free basis to BMJ Publishing Group Ltd ("BMJ") its licensees and where the relevant Journal is co-owned by BMJ to the co-owners of the Journal, to publish the Work in this journal and any other BMJ products and to exploit all rights, as set out in our licence.

The Submitting Author accepts and understands that any supply made under these terms is made by BMJ to the Submitting Author unless you are acting as an employee on behalf of your employer or a postgraduate student of an affiliated institution which is paying any applicable article publishing charge ("APC") for Open Access articles. Where the Submitting Author wishes to make the Work available on an Open Access basis (and intends to pay the relevant APC), the terms of reuse of such Open Access shall be governed by a Creative Commons licence – details of these licences and which Creative Commons licence will apply to this Work are set out in our licence referred to above.

Other than as permitted in any relevant BMJ Author's Self Archiving Policies, I confirm this Work has not been accepted for publication elsewhere, is not being considered for publication elsewhere and does not duplicate material already published. I confirm all authors consent to publication of this Work and authorise the granting of this licence.

For peer review only

Title: WASH-related violence and depressive symptoms in adolescent girls and young women in HIV prevention trials network 068

Authors: Ruvani T. Jayaweera[1*], Dana Goin[2], Rhian Twine[3], Torsten B. Neilands[2], Ryan G. Wagner[3], Sheri A. Lippman[2], Kathleen Kahn[3], Audrey Pettifor[4], Jennifer Ahern[5]

1. Ibis Reproductive Health; Oakland, California, United States of America
2. University of California, San Francisco; San Francisco, California, United States of America
3. University of the Witwatersrand; Johannesburg, South Africa
4. University of North Carolina at Chapel Hill; Chapel Hill, North Carolina, United States of America
5. University of California, Berkeley; Berkeley, California, United States of America

***Corresponding author**:

Ruvani T. Jayaweera
Ibis Reproductive Health
1736 Franklin Street, Suite 600
Oakland, CA 9416
Phone: +1-510-822-2670
Email: rjayaweera@ibisreproductivehealth.org

Keywords: non-partner violence, gender-based violence, violence against women, water sanitation and hygiene, depression

Word count: 2998/4000

For peer review only

ABSTRACT

Objective

There is a lack of research on experiences of WASH-related violence. This study aims to quantify the association between experience or worry of violence when using the toilet or collecting water and depressive symptoms among a cohort of young women in South Africa.

Methods

Data are from visit 3 of the HPTN 068 cohort of adolescent girls in rural Mpumalanga Province, South Africa. 1,870 participants are included in this analysis and were aged 13 – 21 at baseline. Lifetime experience of violence or fear of violence when using the toilet and collecting water was collected by self-report; depressive symptoms in the past week were measured using the Center for Epidemiological Studies Depression Scale (CES-D). We used g-computation to calculate the prevalence difference (PD) and prevalence ratio of depression (CES-D score > 15) associated with each domain of violence, controlling for baseline covariates.

Findings

A total of 15.1% of respondents reported experiencing violence when using the toilet; 17.0% reported experiencing violence when collecting water, and 26.6% reported depression. In adjusted models, those who reported experiencing violence when using the toilet had an 18.3% higher prevalence of depression (95% CI: 13.3%, 25.1%) than those who did not experience violence when using the toilet. Adjusted prevalence of depression was also higher among those who reported violence when collecting water (PD 12.5%, 95% CI: 6.9%, 18.0%), who worried about violence when using the toilet (PD 17.9%, 95% CI: 12.4%, 23.5%, and who worried about violence when collecting water (PD 10.1%, 95% CI: 4.9%, 15.6%), as compared to those who did not report these experiences.

Conclusion

Experience of WASH-related violence is common among young women in rural South Africa, and experience or worry of experiencing violence is associated with higher prevalence of depressive symptoms.

Strengths and Limitations

- This is the first study to quantitatively assess the effect of experiencing violence or fear of experiencing violence on mental health
- Strengths include a large study design, and detailed questions on direct experiences of WASH-related violence and the type of WASH-related activity where the violence occurred
- Additional strengths include a rigorous analytic approach to quantify the absolute marginal difference in prevalence of depression, adjusting for covariates
- Limitations are possible under-reporting of violence as linked to sanitation experiences given the sensitivities around experience of violence, particularly sexual violence

For peer review only - http://bmjopen.bmj.com/site/about/guidelines.xhtml

INTRODUCTION

Despite long-standing global efforts, over 2.1 billion people around the world lack access to safe water and 2.4 billion lack access to safely managed sanitation.[1] Much of the existing research on water, sanitation, and hygiene (WASH) has focused on pathogen-related risks to health or long-term health impacts of infectious diseases.[1] However, this does not capture the breadth of health consequences that arise due to challenges accessing and meeting daily WASH needs. The experience of physical or sexual violence when traveling to or using water and sanitation facilities (WASH-related violence) is an important and under-examined WASH health issue. This paper seeks to quantify the burden of exposure to WASH-related violence and explore its relationship to mental health in a cohort of young women and adolescent girls in South Africa.

Recent studies have noted the increased vulnerability to violence that women and girls face when meeting their daily WASH needs.[2, 3] Around the world, women and girls hold primary responsibility for meeting their household's daily water needs.[2] Coupled with the additional challenge of managing menstruation, and additional stigma faced by women for open defecation, women and girls are most impacted by lack of access to WASH services.[3] A literature review found many examples from humanitarian case studies and practitioner reports that document the heightened risk of sexual violence faced when using communal latrines or practicing open defecation, particularly at night, as well as the heightened risk of sexual violence when traveling to collect water.[3] In addition to the risk of sexual violence, experience of physical violence (for example, fights over resources when queuing for water, intimate partner violence over inability to meet the household's daily water needs) was also identified as a significant public health issue.[3]

A growing body of qualitative research has further elaborated the ways in which shared water and sanitation facilities are a potential site of violence against women.[4] Qualitative research from Uganda, Kenya, and India have found that fear of sexual or physical assault and lack of privacy as driving factors in women's sanitation practices and an important source of anxiety and stress.[2, 5, 6] Quantitative evidence on the linkages between WASH and experiences of violence from India,[7] South Africa,[8] and Kenya[9] have found that women who use open defecation or shared toilet facilities have higher odds of reporting non-partner sexual violence when compared to women with a household toilet. An ecological study found a positive association between the number of sanitation facilities and the rate of reported sexual assaults in a township in South Africa.[10] However, none of these studies assessed whether women experienced violence while they were accessing or using WASH facilities.

While violence is a critical public health and human rights issue on its own, there are also consequent effects of violence on other aspects of health and well-being. While the immediate health impacts of poor access to water and sanitation facilities, such as exposure to water-borne pathogens, vaginal and urinary tract infections, malnutrition, dehydration, and hunger,[5] are well-studied, research linking WASH-related violence to additional downstream health impacts is limited. Furthermore, the health impacts of worrying about the threat of violence when accessing water or sanitation facilities remain unexamined. To our knowledge, no studies have explored the impact of violence experienced when accessing or using WASH facilities and mental health.

Given the lack of evidence on direct experiences with violence when accessing water or sanitation facilities and the potential for downstream health impacts, this paper seeks to quantify

For peer review only - http://bmjopen.bmj.com/site/about/guidelines.xhtml

the association between experience or fear of violence when using the toilet or collecting water and depressive symptoms among a cohort of adolescent girls and young women in South Africa.

Summary Box
**What is already known about this subject?**<br>• Women and girls face increased vulnerability to violence when meeting their daily water, sanitation, and hygiene (WASH) needs<br>• Violence against women while meeting their WASH needs (WASH-related violence) is a critically understudied topic<br>• To our knowledge, no epidemiologic studies have explored the impact of violence experienced when accessing or using WASH facilities and mental health.<br><br>**What does this study add?**<br>• This is the first study to quantitatively assess the impact of experiencing or fear of experiencing violence on mental health<br>• We found that experience of WASH-related violence is common among young women in rural South Africa, and experience or worry of experiencing violence is associated with higher risk of depressive symptoms.<br><br>**How might this impact on clinical practice?**<br>• Our findings demonstrate the importance of centering the needs and concerns of women when designing and implemented water, sanitation, and hygiene interventions to ensure that these interventions do not place women at higher risk of experiencing violence<br>• Addressing built environment determinants of depression like WASH-related violence may help improve mental health outcomes as well as other forms of well-being among adolescent girls and young women

For peer review only

METHODS

Study design

Data for this study are from the HIV Prevention Trials Network (HPTN) 068 cohort of adolescent girls and young women in rural Mpumalanga Province, South Africa, a longitudinal cohort established in 2012 to estimate the effect of cash transfers conditional on staying in school on HIV incidence. Participants were eligible to participate if they were between the ages of 13-20 years, enrolled in grades 8-11 at a participating public school, unmarried, not pregnant at the time of enrollment, able to read, had parents or guardians able to open a bank account, and resided in the Medical Research Council (MRC) / Wits University Agincourt Health and Socio-Demographic Surveillance System (AHDSS) study site. The AHDSS study site is in a rural area of Mpumalanga Province, South Africa that is characterized by high HIV prevalence, high poverty, and migration for work.[11] Most households lack access to piped water in their dwellings, and sanitation is rudimentary.[11]

All households with eligible adolescent girls and young women in the study area were recruited. A total of 2,533 participants enrolled and were followed annually for up to 4 years. At each study visit, participants completed interviewer-administered surveys on a wide variety of domains that included economic activities, health behaviors, health knowledge, and attitudes towards social

For peer review only - http://bmjopen.bmj.com/site/about/guidelines.xhtml

norms; sensitive items, such as sexual behavior and mental health were completed by the participant themselves via Audio Computer Assisted Self Interview (ACASI). Participants' heads of households completed surveys about household composition and wealth at each visit. Full details on the study recruitment and procedures, including a full description of the sample[12] and primary trial outcomes[13], have been published elsewhere.

Measures

The primary outcome of interest for this study is depression, assessed using the Center for Epidemiological Studies Depression Scale (CES-D).[14] The CES-D is a 20-item measure that assesses symptoms of depression over the past week, with frequency of experiencing each symptom as rarely/none of the time, some of/a little of the time, occasional or a moderate amount of time, or all of the time. Scores can range from 0 – 60; in keeping with the literature, we used a cut-off of 16 or greater as an indicator of depression.[15]

Our exposures of interest are WASH-related violence, assessed across four domains: experience of violence when collecting water, experience of violence when using the toilet, fear of violence when collecting water, and fear of violence when using the toilet. Direct experience of violence was assessed by the following question: "How often have you experienced violence when collecting water?" and fear of experiencing violence was assessed by the following question: "Do you ever feel concerned or worried about experiencing violence when using the toilet?" Participants were categorized as being exposed to experience of violence or fear of violence if they responded "Just a few times," "Regularly/about once a week," or "Every day," as opposed to "Never."

We used a directed-acyclic graph to identify a minimally sufficient set of literature-based confounders available in our study. Sociodemographic covariates of interest include age at time of survey, maternal education, paternal education, orphan status, household food insecurity, and decile of household capita consumption (assessed via the household survey).

Analysis

Data for this analysis are drawn from visit 3. We limited our analysis to this time point as only 35.5% of the enrolled sample participated in visit 4 given planned study exit due to graduation from high school. While this data is cross-sectional, experiences or fear of violence was assessed as a lifetime measure, and we assume that those experiences precede the depression measure, which evaluates depression symptoms experienced in the past week.

Records with missing data on parental education were treated as a separate category in analysis; missing data on household food insecurity (n = 7), decile of total household per capita expenditure (n = 8), and orphan status (n = 17) were directly extracted from prior household survey visits.

We used g-computation to calculate the predicted marginal risk difference of depression and the predicted marginal prevalence ratio of depression associated with each individual domain of WASH-related violence, adjusting for age at time of visit, maternal education, paternal education, orphan status, household food insecurity, decile of household capita consumption, and trial arm, and accounting for clustering by village. The nonparametric cluster bootstrap was used to calculate 95% Wald-type percentile-based confidence intervals from 500 resamples.

All analyses were performed using Stata 15 [16].

For peer review only - http://bmjopen.bmj.com/site/about/guidelines.xhtml

Ethics

Ethical approval for this study was obtained by from the University of North Carolina at Chapel Hill (#10–1868), the University of the Witwatersrand Human Research Ethics Committee (#101012), and the Mpumalanga Province's Health Research and Ethics Committee. Approval for analytical work was also obtained by the University of California, Berkeley, and the University of California, San Francisco.

Patient and Public Involvement

Patients and the public were not involved in the design, or conduct, or reporting, or dissemination plans of our research.

RESULTS

A total of 1,870 participants completed a survey at visit 3. Observations were excluded from analysis if they did not complete the CES-D items or the WASH-related violence questions (n = 61, 3.3%). Four participants were additionally excluded as they did not have any data on orphan status at any time point, yielding a final analytic sample of 1,805 observations.

Table 1 displays the sociodemographic characteristics of participants at visit 3. Participants ranged in age from 14 – 22 years; most (79.0%) were between the ages of 16 – 19 at visit 3. One-third (33.6%) of participants had at least one deceased parent, and 7.9% lived in households that reported food insecurity in the past 30 days. The overall prevalence of depression in the sample at visit 3 was 26.6%. A total of 19.3% of participants reported ever being worried about experiencing violence when using the toilet; 16.5% reported being worried about experiencing violence when collecting water. Directly experiencing violence when using the toilet was reported by 15.1% of participants at last recorded visit; experiencing violence when collecting water was reported by 17.0%. A combined total of 26.1% reported directly experiencing violence when using the toilet or collecting water, and 29.1% reported ever being worried about experiencing violence when using the toilet or collecting water. There were no meaningful differences in distribution of the outcome or exposures across sociodemographic covariates of interest except for age; older participants were more likely to report depressive symptoms, experience with WASH-related violence, or fear of WASH-related violence ($p<.001$).

For peer review only

For peer review only - http://bmjopen.bmj.com/site/about/guidelines.xhtml

Table 1. Demographic Characteristics and Prevalence of Depression, Experience with WASH-related Violence, and Fear of WASH-related Violence (N = 1805)

			Depressive Symptoms[1]	Direct Experience of WASH violence[1]	Fear of WASH Violence[1]
**Covariate**	**n**	**%**	**%**	**%**	**%**
Age at Visit					
14	2	0.1	0	50	50
15	260	14.4	17.3	21.2	19.6
16	451	25	24.6	25.9	27.1
17	460	25.5	25.9	24.3	29.1
18	342	18.9	32.5	27.2	33.6
19	173	9.6	28.3	27.7	34.7
20	70	3.9	37.1	37.1	37.1
21	30	1.7	36.7	33.3	30
22	17	0.9	47.1	52.9	47.1
Orphan status					
1 parent deceased	494	27.4	27.7	24.5	26.1
2 parents deceased	112	6.2	26.8	25	27.7
Maternal education					
No school	290	16.1	27.6	25.2	28.6
Some primary	320	17.7	29.7	28.7	31.3
Completed primary	79	4.4	25.3	31.6	31.6
Some high school	515	28.5	26.4	26	30.7
Completed high school	388	21.5	23.7	25.3	27.6
University of vocational	59	3.3	15.3	16.9	22
Unknown/Missing	154	8.5	31.2	25.3	26
Paternal education					
No school	296	16.4	28.4	26.7	31.1
Some primary	246	13.6	28.9	30.5	36.2
Completed primary	80	4.4	26.3	20	23.8
Some high school	340	18.8	22.4	26.5	28.5
Completed high school	427	23.7	26.5	25.3	28.1
University of vocational	73	4	20.5	20.5	20.5
Unknown/Missing	343	19	29.2	25.7	27.4
Household food insecurity					
No	1662	92.1	26.2	26.1	29.1
Yes	143	7.9	30.8	26.6	29.4
Decile of total household per capita expenditures					
1	182	10.1	26.9	24.7	30.2

For peer review only - http://bmjopen.bmj.com/site/about/guidelines.xhtml

2	178	9.9	29.8	28.7	36
3	184	10.2	27.7	28.8	29.3
4	176	9.8	31.3	25.6	30.1
5	176	9.8	27.3	24.4	31.3
6	183	10.1	23.5	21.9	26.8
7	177	9.8	24.3	23.2	23.2
8	182	10.1	27.5	25.3	23.6
9	185	10.2	25.4	29.7	30.8
10	182	10.1	22.5	28.6	30.2
Trial Arm					
1	872	48.3	27.3	28.3	31.7
2	933	51.7	25.9	24	26.8
**Total**	**1805**	**100**	**26.6**	**26.1**	**29.1**

[1] Proportion in each sociodemographic subcategory reporting depressive symptoms, any direct experience of WASH-related violence (while collecting water or using the toilet), or any worry of WASH-related violence (while collecting water or using the toilet)

For peer review only

G-computation results from estimating the absolute and relative association of each exposure on the prevalence of depression (CES-D >= 16) can be found in Table 2. All WASH-related violence exposures were associated with higher prevalence of depression, adjusting for age at time of visit, maternal education, paternal education, orphan status, household food insecurity, decile of household capita consumption, and trial arm. Experiencing violence when using the toilet was associated with a 1.77 times higher prevalence of depression (95% CI: 1.47, 2.05); experiencing violence when collecting water was associated with a 1.51 times higher prevalence of depression (95% CI: 1.25, 1.76); fear of experiencing violence when using the toilet was associated with a 1.77 (95% CI: 1.51, 2.06) times higher prevalence of depression; and fear of experiencing violence when collecting water was associated with a 1.41 times higher prevalence of depression (95% CI: 1.13, 1.66).

Table 2. Adjusted Prevalence Ratios and Prevalence Differences for Depression across Four WASH-related Violence Exposures (N = 1805)

Exposure[1]	Prevalence Ratio (95% CI)[2]	Prevalence Difference[2]
Experienced violence when using the toilet	1.77 (95% CI: 1.47, 2.05)	18.3% (95% CI: 13.3%, 25.1%)
Experienced violence when collecting water	1.51 (95% CI: 1.25, 1.76)	12.5% (95% CI: 6.9%, 18.0%)
Worried of experiencing violence when using the toilet	1.77 (95% CI: 1.51, 2.06)	17.9% (95% CI: 12.4%, 23.5%)
Worried of experiencing violence when collecting water	1.41 (95% CI: 1.13, 1.66)	10.1% (95% CI: 4.9%, 15.6%)

[1] Reference group for each exposure is NO experience with violence (or NO worry of experiencing violence)

[2] G-computation estimates are from four separate logistic regression models adjusting for age at time of visit, maternal education, paternal education, orphan status, household food insecurity, decile of household capita consumption, and trial arm, with 95% CI's calculated from the nonparametric cluster bootstrap.

WASH-related violence was also associated with a substantial 10 – 20% higher prevalence of depression on the absolute scale. After adjusting for covariates, experiencing violence when using the toilet (Prevalence Difference (PD): 18.3; 95% CI: 13.3, 25.1), experiencing violence when collecting water (PD: 12.5, 95% CI: 6.9, 18.0), fear of experiencing violence when using the toilet (PD: 17.9; 95% CI: 12.4, 23.5), and fear of experiencing violence when collecting water (PD: 10.1; 95% CI: 4.9, 15.6) were all associated with higher prevalence of depression. In other words, the prevalence of depression among those who reported experience with violence when using the toilet was 18.1% higher on the absolute scale than the prevalence of depression among those who did not report any experience with violence when using the toilet.

DISCUSSION

This study is among the first to consider how direct experience of WASH-related violence may affect mental health. Directly experiencing violence when using the toilet or collecting water was associated with substantially higher prevalence of depression; additionally, the fear of experiencing violence when using the toilet or collecting water was also associated with a higher prevalence of depression. This analysis builds upon previous qualitative work and practitioner reports, and identifies sanitation and hygiene facilities as a structural/built environment determinant of violence against women and poor mental health outcomes.

For peer review only - http://bmjopen.bmj.com/site/about/guidelines.xhtml

While no studies have quantitatively assessed the burden of WASH-related violence, a recent study using Demographic and Health Survey data from 20 countries in Africa found that 6.2% of women reported non-partner sexual or physical violence in the previous 12 months;[17] a systematic review from 2014 estimated that the lifetime prevalence of experiencing sexual or physical violence from a non-partner is as high as 20% for women living in central Africa.[18] Given the relatively high prevalence of experiencing violence when using the toilet and collecting water in this sample (15.1% and 17.0%, respectively), our analysis suggests that water and sanitation facilities may play a substantial role in the experience of non-partner violence and may be an important site of prevention. Indeed, much of the focus on prevention of violence against women is centered on individual, household, and community-level risk factors; our findings further support calls made by others[17] to consider the role of the built environment, and specifically the design of water and sanitation facilities, as an important factor that may influence non-partner violence against women.

Additionally, our finding that direct experiences of violence when collecting water or using the toilet is associated with depression are aligned with the broader literature on the links between gender-based violence (perpetrated by intimate partners or non-partners) and depression. While the vast majority of the literature on mental health and gender-based violence focuses on intimate-partner violence,[19] what limited evidence does exist on non-partner violence has found that non-partner sexual violence is linked to two-to-three fold increases in odds of depressive symptoms.[20-22] Violence against women is an important human rights issue and has been well documented as having substantial mental health impacts.[23] Depressive symptoms have been linked to worse health and socioeconomic outcomes, including HIV incidence, in this cohort.[24] Thus, addressing built environment determinants of depression like WASH-related violence may help improve mental health outcomes as well as other forms of well-being among adolescent girls and young women. Our findings build on this literature and specifically identify the experience of physical and sexual violence in the context of meeting WASH needs as having the potential to impact mental health.

Beyond direct experiences of violence, qualitative findings in the WASH literature have highlighted the role that fear or worry about experiencing violence—which in itself is a form of violence—may play in poor mental health outcomes. Constant stress and worry around the essential and daily activities of collecting water and using the toilet, as well as mitigating actions to minimize perceived exposure to violence, may be relevant contributors to psychosocial stress[25] which is associated with many negative health outcomes, including depression.[26] Studies globally have highlighted how navigating sanitation facilities access contributes to psychosocial stress through fears of experiencing physical or sexual violence, particularly when needing to use shared latrines or open defecation at night.[5, 6, 25, 27, 28] Findings from this study corroborate what has been found in these qualitative studies; and quantify the substantial extent of concerns and worry about experiencing violence in the context of WASH in women's lives, and its consequent impact on mental health.

This study is not without limitations. Given the sensitivities around experience of violence, particularly sexual violence, there is likely under-reporting of violence as linked to sanitation experiences.[29] However, the ACASI format of the survey was used to minimize underreporting.[30] An additional limitation is the cross-sectional nature of the study: while we assume a temporal relationship between exposure to WASH-related violence and reported symptoms of depression, it is possible that some individuals had a recent experience with

For peer review only - http://bmjopen.bmj.com/site/about/guidelines.xhtml

WASH-related violence that overlapped with the time period in which depressive symptoms were assessed. The 20-item CESD-20 has been validated among students in South Africa,[31] though it has not been validated in this specific population. Furthermore, it is possible that the selected sociodemographic covariates do not fully control for confounding between our exposures and outcomes of interest; for example, poor parental physical and mental health, which were not recorded in this study, have been shown to be associated with higher depression among adolescents, and may also play a role in adolescent exposure to WASH-related violence via impacting the role of the adolescent in water collection for the household. Additionally, school enrollment has been found to be protective against depression among youth in Africa[32] and those who face greater challenges in meeting their WASH-related needs may also be less likely to be enrolled in school; given this study is drawn from a cohort of young women enrolled in school, those who are depressed and experience WASH-violence may be underrepresented in this study, and the potential relationship between WASH-related violence and depression may be much higher. Finally, we lacked data on the location of where direct experiences of WASH-related violence or worries about WASH-related violence took place. Future studies should explore in greater detail whether experiences with WASH-related violence take place near their homes, while at school, or in other places in order to identify key sites for intervention.

The research to-date demonstrates the importance of centering the needs and concerns of women when designing and implemented water, sanitation, and hygiene interventions. Though outside the scope of this paper, the needs of additional groups that may face further marginalization/risk of violence should also be considered, such as elderly people as well as transgender and gender expansive people who likely face greater threat of violence – additional research is warranted to better understand the scope of these individuals' experiences in order to direct resources for prevention.

This study contributes to our understanding of the scope of WASH-related violence experienced by adolescent girls and young women in Mpumalanga, South Africa, and is the first quantitative study to our knowledge that documents the relationship between exposure to WASH-related violence and mental health. Experience of WASH-related violence is common among young women in rural South Africa, and experiencing, or worry of experiencing, violence is associated with substantially higher prevalence of depressive symptoms. Additional research to explore the experience of violence faced by women and girls when collecting water or using the toilet is needed.

For peer review only

Acknowledgements

We thank the HIV Prevention Trials Network 068 study team and all trial participants.

Authors Contribution

AP, RT, RGW, KK, JA, and SAL contributed to the overall study conceptualization, design, and implementation of data collection. RTJ, DG, and JA conceived of the analytic plan with input from TBN, SAL, and AP. RTJ conducted the quantitative analysis. RTJ lead the writing of the manuscript, with contributions, review, and approval from all authors. All authors read and approved the final manuscript. RTJ and JA were responsible for the decision to submit the manuscript.

Competing Interests

The authors have no competing interests to declare.

Funding

This study was funded by the National Institutes of Health (grant R01 MH110186) and by the National Institute of Allergy and Infectious Diseases, the National Institute of Mental Health, and the National Institute on Drug Abuse (awards UM1 AI068619 (HPTN Leadership and Operations Center), UM1AI068617 (HIV Prevention Trials Network Statistical and Data Management Center), and UM1AI068613 (HIV Prevention Trials Network Laboratory Center)). The content is solely the responsibility of the authors and does not necessarily represent the official views of the National Institutes of Health. This work was also supported by the National Institute of Mental Health (grant R01MH087118) and the Carolina Population Center (grant P2C HD050924). Additional support was provided by the Division of Intramural Research, National Institute of Allergy and Infectious Diseases. The MRC/Wits Rural Public Health and Health Transitions Research Unit and Agincourt Health and Socio-Demographic Surveillance System, a node of the South African Population Research Infrastructure Network (SAPRIN), have been supported by the National Department of Science and Innovation, the University of the Witwatersrand, and the Medical Research Council, South Africa, and by the Wellcome Trust, United Kingdom (grants 058893/Z/99/A, 069683/Z/02/Z, 085477/Z/08/Z, and 085477/B/08/Z).

Data Sharing

No additional data available.

For peer review only

REFERENCES

1. World Health Organization. Progress on drinking water, sanitation and hygiene: 2017 update and SDG baselines. 2017.
2. Pommells M, Schuster-Wallace C, Watt S, et al. Gender Violence as a Water, Sanitation, and Hygiene Risk: Uncovering Violence Against Women and Girls as It Pertains to Poor WaSH Access. Violence Against Women. 2018;24(15):1851-62.
3. Sommer M, Ferron S, Cavill S, et al. Violence, gender and WASH: spurring action on a complex, under-documented and sensitive topic. Environment and Urbanization. 2015;27(1):105-16.
4. Corburn J, Hildebrand C. Slum Sanitation and the Social Determinants of Women's Health in Nairobi, Kenya. Journal of Environmental and Public Health. 2015 2015/04/28;2015:209505.
5. Winter S, Barchi F, Dzombo MN. Drivers of women's sanitation practices in informal settlements in sub-Saharan Africa: a qualitative study in Mathare Valley, Kenya. International journal of environmental health research. 2018;28(6):609-25.
6. Khanna T, Das M. Why gender matters in the solution towards safe sanitation? Reflections from rural India. Glob Public Health. 2016;11(10):1185-201.
7. Jadhav A, Weitzman A, Smith-Greenaway E. Household sanitation facilities and women's risk of non-partner sexual violence in India. BMC Public Health. 2016;16(1):016-3797.
8. Gibbs A, Reddy T, Khanyile D, et al. Non-partner sexual violence experience and toilet type amongst young (18-24) women in South Africa: A population-based cross-sectional analysis. Glob Public Health. 2020;8:1-7.
9. Winter SC, Barchi F. Access to sanitation and violence against women: evidence from Demographic Health Survey (DHS) data in Kenya. Int J Environ Health Res. 2016;26(3):291-305.
10. Gonsalves GS, Kaplan EH, Paltiel AD. Reducing sexual violence by increasing the supply of toilets in Khayelitsha, South Africa: a mathematical model. PLoS One. 2015;10(4):e0122244.
11. Kahn K, Collinson MA, Gómez-Olivé FX, et al. Profile: Agincourt health and socio-demographic surveillance system. Int J Epidemiol. 2012;41(4):988-1001.
12. Pettifor A, MacPhail C, Selin A, et al. HPTN 068: A Randomized Control Trial of a Conditional Cash Transfer to Reduce HIV Infection in Young Women in South Africa-Study Design and Baseline Results. AIDS Behav. 2016;20(9):1863-82.
13. Pettifor A, MacPhail C, Hughes JP, et al. The effect of a conditional cash transfer on HIV incidence in young women in rural South Africa (HPTN 068): a phase 3, randomised controlled trial. Lancet Glob Health. 2016;4(12):e978-e88.
14. Radloff LS. The CES-D scale: A self-report depression scale for research in the general population. Applied psychological measurement. 1977;1(3):385-401.
15. Vilagut G, Forero CG, Barbaglia G, et al. Screening for depression in the general population with the Center for Epidemiologic Studies Depression (CES-D): a systematic review with meta-analysis. PLoS One. 2016;11(5):e0155431.
16. LP SC. Stata Statistical Software Release 15: Stata Press Publication; 2017.
17. Barchi F, Winter SC. Non-Partner Violence in Sub-Saharan Africa and the Built Environment: A Multicountry Analysis of the Effects of Sanitation, Water Access, and Urban Settings. Violence Against Women. 2020;26(10):1101-19.
18. Abrahams N, Devries K, Watts C, et al. Worldwide prevalence of non-partner sexual violence: a systematic review. Lancet. 2014;383(9929):1648-54.
19. Beydoun HA, Beydoun MA, Kaufman JS, et al. Intimate partner violence against adult women and its association with major depressive disorder, depressive symptoms and postpartum depression: a systematic review and meta-analysis. Soc Sci Med. 2012;75(6):959-75.
20. Domenech Del Rio I, Sirvent Garcia Del Valle E. Non-partner sexual violence against women in Spain: lifetime prevalence, perpetrators and consequences on mental health. J Public Health. 2017;39(4):738-44.
21. Hankin CS, Skinner KM, Sullivan LM, et al. Prevalence of depressive and alcohol abuse symptoms among women VA outpatients who report experiencing sexual assault while in the military. J Trauma Stress. 1999;12(4):601-12.
22. Kimerling R, Gima K, Smith MW, et al. The Veterans Health Administration and military sexual trauma. Am J Public Health. 2007;97(12):2160-6.

For peer review only - http://bmjopen.bmj.com/site/about/guidelines.xhtml

23. Jordan CE, Campbell R, Follingstad D. Violence and women's mental health: the impact of physical, sexual, and psychological aggression. Annual review of clinical psychology. 2010;6.
24. Goin DE, Pearson RM, Craske MG, et al. Depression and Incident HIV in Adolescent Girls and Young Women in HIV Prevention Trials Network 068: Targets for Prevention and Mediating Factors. Am J Epidemiol. 2019;189(5):422-32.
25. Shiras T, Cumming O, Brown J, et al. Shared latrines in Maputo, Mozambique: exploring emotional well-being and psychosocial stress. BMC Int Health Hum Rights. 2018;18(1):018-0169.
26. Gilman SE, Trinh NH, Smoller JW, et al. Psychosocial stressors and the prognosis of major depression: a test of Axis IV. Psychol Med. 2013;43(2):303-16.
27. Corburn J, Karanja I. Informal settlements and a relational view of health in Nairobi, Kenya: Sanitation, gender and dignity. Health promotion international. 2014;31(2):258-69.
28. Sahoo KC, Hulland KR, Caruso BA, et al. Sanitation-related psychosocial stress: A grounded theory study of women across the life-course in Odisha, India. Soc Sci Med. 2015;139:80-9.
29. Joshi D, Fawcett B, Mannan F. Health, hygiene and appropriate sanitation: experiences and perceptions of the urban poor. Environment and Urbanization. 2011;23(1):91-111.
30. Hewett PC, Mensch BS, Erulkar AS. Consistency in the reporting of sexual behaviour by adolescent girls in Kenya: a comparison of interviewing methods. Sex Transm Infect. 2004;80(suppl 2):ii43-ii8.
31. Pretorius TB. Cross-cultural application of the Center for Epidemiological Studies Depression Scale: a study of black South African students. Psychol Rep. 1991;69(3 Pt 2):1179-85.
32. Kilburn K, Prencipe L, Hjelm L, et al. Examination of performance of the Center for Epidemiologic Studies Depression Scale Short Form 10 among African youth in poor, rural households. BMC psychiatry. 2018;18(1):1-13.

For peer review only

For peer review only - http://bmjopen.bmj.com/site/about/guidelines.xhtml

STROBE Statement—Checklist of items that should be included in reports of ***cohort studies***

	Item No	Recommendation	Page No
**Title and abstract**	1	(*a*) Indicate the study's design with a commonly used term in the title or the abstract	1
		(*b*) Provide in the abstract an informative and balanced summary of what was done and what was found	2
**Introduction**			
Background/rationale	2	Explain the scientific background and rationale for the investigation being reported	3
Objectives	3	State specific objectives, including any prespecified hypotheses	3-4
**Methods**			
Study design	4	Present key elements of study design early in the paper	4
Setting	5	Describe the setting, locations, and relevant dates, including periods of recruitment, exposure, follow-up, and data collection	4-5
Participants	6	(*a*) Give the eligibility criteria, and the sources and methods of selection of participants. Describe methods of follow-up	4-5
		(*b*) For matched studies, give matching criteria and number of exposed and unexposed	
Variables	7	Clearly define all outcomes, exposures, predictors, potential confounders, and effect modifiers. Give diagnostic criteria, if applicable	5
Data sources/ measurement	8*	For each variable of interest, give sources of data and details of methods of assessment (measurement). Describe comparability of assessment methods if there is more than one group	5
Bias	9	Describe any efforts to address potential sources of bias	5
Study size	10	Explain how the study size was arrived at	4
Quantitative variables	11	Explain how quantitative variables were handled in the analyses. If applicable, describe which groupings were chosen and why	5
Statistical methods	12	(*a*) Describe all statistical methods, including those used to control for confounding	5
		(*b*) Describe any methods used to examine subgroups and interactions	n/a
		(*c*) Explain how missing data were addressed	5
		(*d*) If applicable, explain how loss to follow-up was addressed	5
		(*e*) Describe any sensitivity analyses	n/a
**Results**			
Participants	13*	(a) Report numbers of individuals at each stage of study—eg numbers potentially eligible, examined for eligibility, confirmed eligible, included in the study, completing follow-up, and analysed	6
		(b) Give reasons for non-participation at each stage	6
		(c) Consider use of a flow diagram	n/a
Descriptive data	14*	(a) Give characteristics of study participants (eg demographic, clinical, social) and information on exposures and potential confounders	6-7
		(b) Indicate number of participants with missing data for each variable of interest	6
		(c) Summarise follow-up time (eg, average and total amount)	
Outcome data	15*	Report numbers of outcome events or summary measures over time	6-7

For peer review only

For peer review only - http://bmjopen.bmj.com/site/about/guidelines.xhtml

Main results	16	(*a*) Give unadjusted estimates and, if applicable, confounder-adjusted estimates and their precision (eg, 95% confidence interval). Make clear which confounders were adjusted for and why they were included	6-8
		(*b*) Report category boundaries when continuous variables were categorized	n/a
		(*c*) If relevant, consider translating estimates of relative risk into absolute risk for a meaningful time period	8
Other analyses	17	Report other analyses done—eg analyses of subgroups and interactions, and sensitivity analyses	
**Discussion**			
Key results	18	Summarise key results with reference to study objectives	8
Limitations	19	Discuss limitations of the study, taking into account sources of potential bias or imprecision. Discuss both direction and magnitude of any potential bias	9-10
Interpretation	20	Give a cautious overall interpretation of results considering objectives, limitations, multiplicity of analyses, results from similar studies, and other relevant evidence	10
Generalisability	21	Discuss the generalisability (external validity) of the study results	10
**Other information**			
Funding	22	Give the source of funding and the role of the funders for the present study and, if applicable, for the original study on which the present article is based	11

*Give information separately for exposed and unexposed groups.

Note: An Explanation and Elaboration article discusses each checklist item and gives methodological background and published examples of transparent reporting. The STROBE checklist is best used in conjunction with this article (freely available on the Web sites of PLoS Medicine at http://www.plosmedicine.org/, Annals of Internal Medicine at http://www.annals.org/, and Epidemiology at http://www.epidem.com/). Information on the STROBE Initiative is available at http://www.strobe-statement.org.

For peer review only

For peer review only - http://bmjopen.bmj.com/site/about/guidelines.xhtml

BMJ Open

Associations between WASH-related violence and depressive symptoms in adolescent girls and young women in South Africa (HPTN 068): a cross-sectional analysis

Journal:	*BMJ Open*
Manuscript ID	bmjopen-2022-061032.R1
Article Type:	Original research
Date Submitted by the Author:	17-May-2022
Complete List of Authors:	Jayaweera, Ruvani T.; Ibis Reproductive Health<br>Goin, Dana; University of California San Francisco, Obstetrics, Gynecology, & Reproductive Sciences<br>Twine, Rhian; University of the Witwatersrand,<br>Neilands, Torsten; University of California San Francisco<br>Wagner, Ryan; University of the Witwatersrand<br>Lippman, Sheri ; University of California San Francisco<br>Kahn, Kathleen; University of the Witwatersrand,<br>Pettifor, Audrey; University of North Carolina at Chapel Hill Gillings School of Global Public Health, Epidemiology<br>Ahern, Jennifer; University of California Berkeley School of Public Health, Division of Epidemiology
**Primary Subject Heading**:	Global health
Secondary Subject Heading:	Epidemiology, Mental health, Public health
Keywords:	EPIDEMIOLOGY, Depression & mood disorders < PSYCHIATRY, PUBLIC HEALTH, Community child health < PAEDIATRICS, MENTAL HEALTH

SCHOLARONE™
Manuscripts

For peer review only - http://bmjopen.bmj.com/site/about/guidelines.xhtml

BMJ

I, the Submitting Author has the right to grant and does grant on behalf of all authors of the Work (as defined in the below author licence), an exclusive licence and/or a non-exclusive licence for contributions from authors who are: i) UK Crown employees; ii) where BMJ has agreed a CC-BY licence shall apply, and/or iii) in accordance with the terms applicable for US Federal Government officers or employees acting as part of their official duties; on a worldwide, perpetual, irrevocable, royalty-free basis to BMJ Publishing Group Ltd ("BMJ") its licensees and where the relevant Journal is co-owned by BMJ to the co-owners of the Journal, to publish the Work in this journal and any other BMJ products and to exploit all rights, as set out in our licence.

The Submitting Author accepts and understands that any supply made under these terms is made by BMJ to the Submitting Author unless you are acting as an employee on behalf of your employer or a postgraduate student of an affiliated institution which is paying any applicable article publishing charge ("APC") for Open Access articles. Where the Submitting Author wishes to make the Work available on an Open Access basis (and intends to pay the relevant APC), the terms of reuse of such Open Access shall be governed by a Creative Commons licence – details of these licences and which Creative Commons licence will apply to this Work are set out in our licence referred to above.

Other than as permitted in any relevant BMJ Author's Self Archiving Policies, I confirm this Work has not been accepted for publication elsewhere, is not being considered for publication elsewhere and does not duplicate material already published. I confirm all authors consent to publication of this Work and authorise the granting of this licence.

For peer review only

Title: Associations between WASH-related violence and depressive symptoms in adolescent girls and young women in South Africa (HPTN 068): a cross-sectional analysis

Authors: Ruvani T. Jayaweera[1*], Dana E. Goin[2], Rhian Twine[3], Torsten B. Neilands[2], Ryan G. Wagner[3], Sheri A. Lippman[2], Kathleen Kahn[3], Audrey Pettifor[4], Jennifer Ahern[5]

1. Ibis Reproductive Health; Oakland, California, United States of America
2. University of California, San Francisco; San Francisco, California, United States of America
3. University of the Witwatersrand; Johannesburg, South Africa
4. University of North Carolina at Chapel Hill; Chapel Hill, North Carolina, United States of America
5. University of California, Berkeley; Berkeley, California, United States of America

***Corresponding author**:

Ruvani T. Jayaweera
Ibis Reproductive Health
1736 Franklin Street, Suite 600
Oakland, CA 9416
Phone: +1-510-822-2670
Email: rjayaweera@ibisreproductivehealth.org

Keywords: non-partner violence, gender-based violence, violence against women, water sanitation and hygiene, depression

Word count: 3410/4000

For peer review only - http://bmjopen.bmj.com/site/about/guidelines.xhtml

ABSTRACT

Objective

There is a lack of research on experiences of WASH-related violence. This study aims to quantify the association between experience or worry of violence when using the toilet or collecting water and depressive symptoms among a cohort of young women in South Africa.

Methods

Data are from visit 3 of the HPTN 068 cohort of adolescent girls in rural Mpumalanga Province, South Africa. Participants (N=1,798) included in this analysis were aged 13 – 21 at baseline. Lifetime experience of violence or fear of violence when using the toilet and collecting water was collected by self-report; depressive symptoms in the past week were measured using the Center for Epidemiological Studies Depression Scale (CES-D). We used G-computation to calculate the prevalence difference (PD) and prevalence ratio of depression (CES-D score > 15) associated with each domain of violence, controlling for baseline covariates.

Findings

A total of 15.1% of respondents reported experiencing violence when using the toilet; 17.1% reported experiencing violence when collecting water, and 26.7% reported depression. In adjusted models, those who reported experiencing violence when using the toilet had an 18.1% higher prevalence of depression (95% CI: 11.6%, 24.4%) than those who did not experience violence when using the toilet. Adjusted prevalence of depression was also higher among those who reported violence when collecting water (PD 11.9%, 95% CI: 6.7%, 17.2%), and who worried about violence when using the toilet (PD 12.8%, 95% CI: 7.9%, 19.8%), as compared to those who did not report these experiences. Worrying about violence when collecting water was not associated with depression after adjusting for covariates.

Conclusion

Experience of WASH-related violence is common among young women in rural South Africa, and experience or worry of experiencing violence is associated with higher prevalence of depressive symptoms.

Strengths and Limitations

- This is the first study to quantitatively assess the effect of experiencing violence or fear of experiencing violence on mental health
- Strengths include a large study design, and detailed questions on direct experiences of WASH-related violence and the type of WASH-related activity where the violence occurred
- Additional strengths include a rigorous analytic approach to quantify the absolute marginal difference in prevalence of depression, adjusting for covariates
- Limitations are the cross-sectional nature of the study, and possible under-reporting of violence as linked to sanitation experiences given the sensitivities around experience of violence, particularly sexual violence

For peer review only - http://bmjopen.bmj.com/site/about/guidelines.xhtml

INTRODUCTION

Despite long-standing global efforts, approximately two billion people around the world lack access to safe water and 3.6 billion lack access to safely managed sanitation in 2020.[1] Much of the existing public health research on water, sanitation, and hygiene (WASH) has focused on pathogen-related risks to health or long-term health impacts of infectious diseases.[2] However, this does not capture the breadth of health consequences that arise due to challenges accessing and meeting daily WASH needs. While recent studies have noted the increased vulnerability to violence that women and girls face when meeting their daily WASH needs[3, 4], the experience of physical or sexual violence when traveling to or using water and sanitation facilities (WASH-related violence) remains an under-examined issue. This paper seeks to quantify the burden of exposure to WASH-related violence and explore its relationship to mental health in a cohort of young women and adolescent girls in South Africa.

Around the world, women and girls hold primary responsibility for meeting their household's daily water needs.[3] Coupled with the additional challenge of managing menstruation, and additional stigma faced by women for open defecation, women and girls are most impacted by lack of access to WASH services.[4] A literature review highlighted many examples from humanitarian case studies and practitioner reports that document the heightened risk of sexual violence faced when using communal latrines or practicing open defecation, particularly at night, as well as the heightened risk of sexual violence when traveling to collect water.[4] In addition to the risk of sexual violence, experience of physical violence (for example, fights over resources when queuing for water, intimate partner violence over inability to meet the household's daily water needs) was also identified as a significant public health issue.[4]

A growing body of research has further elaborated the ways in which shared water and sanitation facilities are a potential site of violence against women.[5] Qualitative research from Uganda, Kenya, and India have found that fear of sexual or physical assault and lack of privacy as driving factors in women's sanitation practices and an important source of anxiety and stress.[3, 6-8] Quantitative evidence on the linkages between WASH and experiences of violence from India,[9, 10] South Africa,[11] and Kenya[12] have found that women who use open defecation or shared toilet facilities have higher odds of reporting non-partner sexual violence when compared to women with a household toilet. An analysis of Demographic and Health survey data from 25 African countries found that unimproved water and sanitation facilities was associated with higher odds of experiencing intimate partner violence.[13] An ecological study found a positive association between the number of sanitation facilities and the rate of reported sexual assaults in a township in South Africa.[14] However, none of these studies assessed whether women experienced violence while they were accessing or using WASH facilities.

While violence is a critical public health and human rights issue on its own, there are also consequent effects of violence on other aspects of health and well-being. While the immediate health impacts of poor access to water and sanitation facilities, such as exposure to water-borne pathogens, vaginal and urinary tract infections, malnutrition, dehydration, and hunger,[6] are well-studied, research linking WASH-related violence to additional downstream health impacts is limited. However, new measures of water[15] and sanitation[16] insecurity include concerns about violence as a domain in these measures, and there is a growing body of research linking water and sanitation insecurity with poor mental health outcomes.[17-19] However, to our

For peer review only - http://bmjopen.bmj.com/site/about/guidelines.xhtml

knowledge, no studies have quantitatively explored the relationship between direct experiences of violence experienced while accessing or using WASH facilities on mental health.

Given the lack of evidence on direct experiences with violence when accessing water or sanitation facilities and the potential for downstream health impacts, this paper seeks to quantify the association between experience or fear of violence when using the toilet or collecting water and depressive symptoms among a cohort of adolescent girls and young women in South Africa.

METHODS

Study design

Data for this study are from the HIV Prevention Trials Network (HPTN) 068 cohort of adolescent girls and young women in rural Mpumalanga Province, South Africa, a longitudinal cohort established in 2012 to estimate the effect of cash transfers conditional on staying in school on HIV incidence. Participants were eligible to participate if they were between the ages of 13-20 years, enrolled in grades 8-11 at a participating public school, unmarried, not pregnant at the time of enrollment, able to read, had parents or guardians able to open a bank account, and resided in the Medical Research Council (MRC) / Wits University Agincourt Health and Socio-Demographic Surveillance System (AHDSS) study site. The AHDSS study site is in a rural area of Mpumalanga Province, South Africa that is characterized by high HIV prevalence, high poverty, and migration for work.[20] Most households lack access to piped water in their dwellings, and sanitation is rudimentary.[20]

All households with eligible adolescent girls and young women in the study area were recruited. A total of 2,533 participants enrolled and were followed annually for up to 4 years. At each study visit, participants completed interviewer-administered surveys on a wide variety of domains that included economic activities, health behaviors, health knowledge, and attitudes towards social norms. Sensitive items, such as sexual behavior and mental health, were completed by the participants themselves via Audio Computer Assisted Self Interview (ACASI), where participants listen to questions and response categories through headphones and select their responses. Prior research has found higher reporting of sensitive issues via ACASI as compared to interviewer-administered surveys.[21] Participants' heads of households completed surveys about household composition and wealth at each visit. Full details on the study recruitment and procedures, including a full description of the sample[22] and primary trial outcomes[23], have been published elsewhere.

Measures

The primary outcome of interest for this study is depression, assessed using the Center for Epidemiological Studies Depression Scale (CES-D).[24] The CES-D is a 20-item measure that assesses symptoms of depression over the past week, with frequency of experiencing each symptom as rarely/none of the time, some of/a little of the time, occasional or a moderate amount of time, or all of the time. Scores can range from 0 – 60; in keeping with the literature, we used a cut-off of 16 or greater as an indicator of depression.[25]

Our exposures of interest are WASH-related violence, assessed across four domains: experience of violence when collecting water, experience of violence when using the toilet, fear

For peer review only - http://bmjopen.bmj.com/site/about/guidelines.xhtml

of violence when collecting water, and fear of violence when using the toilet. Direct experience of violence was assessed by the following question: “How often have you experienced violence when collecting water?” and fear of experiencing violence was assessed by the following question: “Do you ever feel concerned or worried about experiencing violence when using the toilet?” Participants were categorized as being exposed to experience of violence or fear of violence if they responded “Just a few times,” “Regularly/about once a week,” or “Every day,” as opposed to “Never.” Though both direct experiences with violence and fear of experiencing violence were assessed at the same time point, based on the wording of the question, we assume that direct experience of violence precedes fear of experiencing violence.

We used a directed-acyclic graph to identify a minimally sufficient set of literature-based confounders available in our study. We identified the following sociodemographic covariates of interest: age at time of survey[11, 12, 26], maternal and paternal education[26], orphan status[26], household food insecurity in the past 30 days[11, 18, 27], decile of household capita consumption[12, 26], and any negative events experienced by the household in the past 12 months[18, 27] (assessed via the household survey). Negative events reported in the household survey included experiences such as death or serious illness of a household member, loss of livestock or crop failure, job loss or loss of government grants, or loss or destruction of property. We also controlled for toilet type[9, 11] and household water source[18].

Analysis

Data for this analysis are drawn from visit 3. We limited our analysis to this time point as only 35.5% of the enrolled sample participated in visit 4 given planned study exit due to graduation from high school. While this data is cross-sectional, experiences or fear of violence was assessed as a lifetime measure, and we assume that those experiences precede the depression measure, which evaluates depression symptoms experienced in the past week. We also assume that experience of violence precedes fear of violence.

Records with missing data on parental education were treated as a separate category in analysis; missing data on household food insecurity (n = 7), decile of total household per capita expenditure (n = 8), and orphan status (n = 17) were directly imputed from prior household survey visits.

We used G-computation to calculate the predicted marginal prevalence difference of depression and the predicted marginal prevalence ratio of depression associated with each individual domain of WASH-related violence. All models adjusted for age at time of visit, maternal education, paternal education, orphan status, household food insecurity, decile of household capita consumption, negative household experiences, and trial arm, and accounted for clustering by village by using nonparametric cluster bootstrap to calculate 95% Wald-type percentile-based confidence intervals from 500 resamples. Models assessing fear of violence when collecting water and fear of violence when using the toilet additionally adjusted for prior experience of violence when collecting water and when using the toilet, respectively. Models assessing experience or fear of violence when collecting water additionally adjusted for household water source; models assessing experience or fear of violence when using the toilet additionally adjusted for household toilet type. All analyses were performed using Stata 15 [28].

Ethics

Ethical approval for this study was obtained by from the University of North Carolina at Chapel Hill (#10–1868), the University of the Witwatersrand Human Research Ethics Committee (#101012), and the Mpumalanga Province's Health Research and Ethics Committee. Approval for analytical work was also obtained by the University of California, Berkeley, and the University of California, San Francisco.

Patient and Public Involvement

All studies, including the HPTN 068 trial, conducted in the AHDSS study site receive permission to undertake research activities from a forum comprised of community and village leaders. Findings from the main trial were communicated to the community via community meetings and factsheets. Additional details on community involvement are available via the study site's Public Engagement Office.[29]

RESULTS

A total of 1,870 participants completed a survey at visit 3. Observations were excluded from analysis if they did not complete the CES-D items or the WASH-related violence questions (n = 61, 3.3%). Participants were additionally excluded as they did not have any data on orphan status or household data on toilet type and water source (n = 11, 0.6%) at any time point, yielding a final analytic sample of 1,798 observations.

Table 1 displays the sociodemographic characteristics of participants at visit 3. Participants ranged in age from 14 – 22 years; most (79.1%) were between the ages of 16 – 19 at visit 3. One-third (33.6%) of participants had at least one deceased parent, and 7.9% lived in households that reported food insecurity in the past 30 days. The overall prevalence of depression in the sample at visit 3 was 26.7%. A total of 19.4% of participants reported ever being worried about experiencing violence when using the toilet; 16.5% reported being worried about experiencing violence when collecting water. Directly experiencing violence when using the toilet was reported by 15.1% of participants; experiencing violence when collecting water was reported by 17.1%. A combined total of 26.2% reported directly experiencing violence when using the toilet or collecting water, and 29.3% reported ever being worried about experiencing violence when using the toilet or collecting water. Age, water source, and toilet type were all associated with depressive symptoms in Pearson chi-square tests of independence adjusted for clustering by village; age and water source were also associated with any experience of violence with WASH-related violence, and age was associated with fear of WASH-related violence.

For peer review only - http://bmjopen.bmj.com/site/about/guidelines.xhtml

For peer review only

Table 1. Demographic Characteristics and Prevalence of Depression, Experience with WASH-related Violence, and Fear of WASH-related Violence (N = 1798)

			Depressive Symptoms[1]	Direct Experience of WASH violence[1]	Fear of WASH Violence[1]
**Covariate**	**n**	**%**	**%**	**%**	**%**
Age at Visit[2]					
14	2	0.1	0	50	50
15	259	14.4	17.4	21.2	19.7
16	449	25.0	24.7	26.1	27.2
17	459	25.5	25.9	24.4	29.2
18	341	19.0	32.6	27.3	33.7
19	173	9.6	28.3	27.7	34.7
20	70	3.9	37.1	37.1	37.1
21	30	1.7	36.7	33.3	30.0
22	15	0.8	53.3	60.0	53.3
Orphan status					
1 parent deceased	493	27.4	27.8	24.5	26.2
2 parents deceased	112	6.2	26.8	25.0	27.7
Maternal education					
No school	288	16.0	27.8	25.3	28.8
Some primary	320	17.8	29.7	28.7	31.3
Completed primary	79	4.4	25.3	31.6	31.6
Some high school	514	28.6	26.5	26.1	30.7
Completed high school	386	21.5	23.8	25.4	27.7
University or vocational	57	3.2	15.8	17.5	22.8
Unknown/Missing	154	8.6	31.2	25.3	26.0
Paternal education					
No school	295	16.4	28.4	26.8	31.2
Some primary	246	13.7	28.9	30.5	36.2
Completed primary	80	4.4	26.3	20.0	23.8
Some high school	339	18.9	22.4	26.5	28.6
Completed high school	425	23.6	26.6	25.4	28.2
University or vocational	71	3.9	21.1	21.1	21.1
Unknown/Missing	342	19.0	29.2	25.7	27.5
Household food insecurity					
No	1656	92.1	26.3	26.1	29.2
Yes	142	7.9	31.0	26.8	29.3
Decile of total household per capita expenditures					
1	181	10.1	27.1	24.9	30.4

For peer review only

For peer review only - http://bmjopen.bmj.com/site/about/guidelines.xhtml

2	178	9.9	29.8	28.7	36.0
3	183	10.2	27.9	29.0	29.5
4	176	9.8	31.3	25.6	30.1
5	176	9.8	27.3	24.4	31.3
6	182	10.1	23.6	22.0	26.9
7	177	9.8	24.3	23.2	23.2
8	181	10.1	27.6	25.4	23.8
9	183	10.2	25.7	30.1	31.1
10	181	10.1	22.7	28.7	30.4
Household experienced any recent negative event					
No	1463	81.4	26.0	26.2	29.2
Yes	335	18.6	29.6	26.3	29.6
Household water source[3]					
Piped water	1188	66.1	24.7	23.6	27.4
Public tap/standpipe	460	25.6	29.8	32.8	34.8
Rain/surface water	15	0.8	20.0	33.3	26.7
Tanker truck	88	4.9	33.0	25	25.0
Well (tube, dug, borehole)	47	2.6	38.3	27.7	29.8
Household toilet type[4]					
Flush or pour toilet	106	5.9	14.2	24.5	33.0
No facility/open defecation	88	4.9	25.0	27.3	29.5
Pit latrine	1604	89.2	27.6	26.2	29.0
Trial Arm[5]					
1	868	48.3	27.3	28.5	31.8
2	930	51.7	25.9	24.1	26.9
**Total**	**1798**	**100**	**26.7**	**26.2**	**29.3**

[1] Proportion in each sociodemographic subcategory reporting depressive symptoms, any direct experience of WASH-related violence (while collecting water or using the toilet), or any worry of WASH-related violence (while collecting water or using the toilet)

[2] p =.001 (age and depressive symptoms), p = .017 (age and any experience of violence), p = .017 (age and any fear of violence)

[3] p = .027 (water source and depressive symptoms), p = .039 (water source and any experience of violence)

[4] p = .010 (toilet type and depressive symptoms)

[5] p = .014 (trial arm and any experience of violence), p = .036 (trial arm and any fear of violence)

For peer review only - http://bmjopen.bmj.com/site/about/guidelines.xhtml

G-computation results from estimating the absolute and relative association of each exposure on the prevalence of depression (CES-D >= 16) can be found in Table 2. After adjusting for covariates, experiencing violence when using the toilet was associated with a 1.76 times higher prevalence of depression (95% CI: 1.47, 2.05) and experiencing violence when collecting water was associated with a 1.48 times higher prevalence of depression (95% CI: 1.26, 1.77). While fear of experiencing violence when collecting water was not associated with a higher prevalence of depression (Prevalence Ratio: 1.05, 95% CI: 0.81, 1.44), fear of experiencing violence when using the toilet was associated with a 1.53 (95% CI: 1.31, 1.92) times higher prevalence of depression, after controlling for covariates (including prior experience of violence when using the toilet).

Table 2. Adjusted Prevalence Ratios and Prevalence Differences for Depression across Four WASH-related Violence Exposures (N = 1,798)

Exposure[1]	Prevalence Ratio (95% CI)[2]	Prevalence Difference[2]
Experienced violence when using the toilet[3]	1.76 (95% CI: 1.47, 2.05)	18.1% (95% CI: 11.6%, 24.4%)
Experienced violence when collecting water[4]	1.48 (95% CI: 1.26, 1.77)	11.9% (95% CI: 6.7%, 17.2%)
Worried of experiencing violence when using the toilet[5]	1.53 (95% CI: 1.31, 1.92)	12.8% (95% CI: 7.9%, 19.8%)
Worried of experiencing violence when collecting water[6]	1.05 (95% CI: 0.81, 1.44)	1.4% (95% CI: -5.3%, 10.9%)

[1] Reference group for each exposure is NO experience with violence (or NO worry of experiencing violence)
[2] G-computation estimates are from four separate logistic regression models adjusting for covariates, with 95% CI's calculated from the nonparametric cluster bootstrap.
[3] Model adjusts for age at time of visit, maternal education, paternal education, orphan status, household food insecurity, decile of household capita consumption, household experience of any negative event, trial arm, and toilet type.
[4] Model adjusts for age at time of visit, maternal education, paternal education, orphan status, household food insecurity, decile of household capita consumption, household experience of any negative event, trial arm, and water source.
[5] Model adjusts for age at time of visit, maternal education, paternal education, orphan status, household food insecurity, decile of household capita consumption, household experience of any negative event, trial arm, toilet type, and experience of violence when using the toilet.
[6] Model adjusts for age at time of visit, maternal education, paternal education, orphan status, household food insecurity, decile of household capita consumption, household experience of any negative event, trial arm, water source, and experience of violence when collecting water.

WASH-related violence was also associated with higher prevalence of depression on the absolute scale for experience of violence when using the toilet and collecting water, and fear of experiencing violence when using the toilet. After adjusting for covariates, experiencing violence when using the toilet (Prevalence Difference (PD): 18.1%; 95% CI: 11.6%, 24.4), experiencing violence when collecting water (PD: 11.9%, 95% CI: 6.7%, 17.2%), and fear of experiencing violence when using the toilet (PD: 12.8%; 95% CI: 7.9%, 19.8%) were all associated with higher prevalence of depression. In other words, the prevalence of depression among those who reported experience with violence when using the toilet was 18.1 percentage points higher on the absolute scale than the prevalence of depression among those who did not report any experience with violence when using the toilet, controlling for covariates.

For peer review only - http://bmjopen.bmj.com/site/about/guidelines.xhtml

DISCUSSION

This study is among the first to consider how direct experience of WASH-related violence may affect mental health. Directly experiencing violence when using the toilet or collecting water was associated with substantially higher prevalence of depression; additionally, the fear of experiencing violence when using the toilet, even when controlling for prior experience of experiencing violence when using the toilet, was also associated with a higher prevalence of depression. This analysis builds upon previous qualitative work and practitioner reports, and identifies water and sanitation facilities as a structural/built environment determinant of violence against women and poor mental health outcomes.

While no studies have quantitatively assessed the burden of WASH-related violence, a recent study using Demographic and Health Survey data from 20 countries in Africa found that 6.2% of women reported non-partner sexual or physical violence in the previous 12 months;[30] a systematic review from 2014 estimated that the lifetime prevalence of experiencing sexual or physical violence from a non-partner is as high as 20% for women living in central Africa.[31] Given the relatively high prevalence of experiencing violence when using the toilet and collecting water in this sample (15.1% and 17.0%, respectively), our analysis suggests that water and sanitation facilities may play a substantial role in the experience of non-partner violence and may be an important site of prevention. Indeed, much of the focus on prevention of violence against women is centered on individual, household, and community-level risk factors; our findings further support calls made by others[30] to consider the role of the built environment, and specifically access to water and sanitation facilities, as an important factor that may influence non-partner violence against women.

Additionally, our finding that direct experiences of violence when collecting water or using the toilet is associated with depression are aligned with the broader literature on the links between gender-based violence (perpetrated by intimate partners or non-partners) and depression. While the vast majority of the literature on mental health and gender-based violence focuses on intimate-partner violence,[32] what limited evidence does exist on non-partner violence has found that non-partner sexual violence is linked to two-to-three fold increases in odds of depressive symptoms.[33-35] Violence against women is an important human rights issue and has been well documented as having substantial mental health impacts.[36] Depressive symptoms have been linked to worse health and socioeconomic outcomes, including HIV incidence, in this cohort.[26] Thus, addressing determinants of depression related to the built environment, such as access to WASH facilities and consequent exposure to WASH-related violence, may help improve mental health outcomes as well as other forms of well-being among adolescent girls and young women. Our findings build on this literature and specifically identify the experience of physical and sexual violence in the context of meeting WASH needs as having the potential to impact mental health.

Beyond direct experiences of violence, findings in the WASH literature, detailed below, have highlighted the role that fear or worry about experiencing violence—which itself is a form of violence—may play in poor mental health outcomes. Constant stress and worry around the essential and daily activities of collecting water and using the toilet, as well as mitigating actions to minimize perceived exposure to violence, may be relevant contributors to psychosocial stress[17-19, 37] which is associated with many negative health outcomes, including depression.[38] Studies globally have highlighted how navigating sanitation facilities access contributes to psychosocial stress through fears of experiencing physical or sexual violence,

For peer review only - http://bmjopen.bmj.com/site/about/guidelines.xhtml

particularly when needing to use shared latrines or open defecation at night.[6, 7, 37, 39, 40] Findings from this study corroborate what has been found in these studies; and quantify the substantial extent of concerns and worry about experiencing violence in the context of WASH in women's lives, even after controlling for prior experience of violence, and its consequent impact on mental health.

This study is not without limitations. Given the sensitivities around experience of violence, particularly sexual violence, there is likely under-reporting of violence as linked to sanitation experiences.[41] However, the ACASI format of the survey was used to minimize underreporting.[21] An additional limitation is the cross-sectional nature of the study: while we assume a temporal relationship between exposure to WASH-related violence and reported symptoms of depression, it is possible that some individuals had a recent experience with WASH-related violence that overlapped with the time period in which depressive symptoms were assessed. The 20-item CESD-20 has been validated among students in South Africa,[42] though it has not been validated in this specific population. Furthermore, it is possible that the selected sociodemographic covariates do not fully control for confounding between our exposures and outcomes of interest; for example, poor parental physical and mental health, which were not recorded in this study, have been shown to be associated with higher depression among adolescents, and may also play a role in adolescent exposure to WASH-related violence via impacting the role of the adolescent in water collection for the household. While we were unable to control for distance to toilet or water source, we hypothesize that the potential confounding effects of these variables are at least partially accounted for by toilet type and water source. Additionally, school enrollment has been found to be protective against depression among youth in Africa[43] and those who face greater challenges in meeting their WASH-related needs may also be less likely to be enrolled in school; given this study is drawn from a cohort of young women enrolled in school, those who are depressed and experience WASH-violence may be underrepresented in this study, and the potential relationship between WASH-related violence and depression may be much higher. Finally, we lacked data on the location of where direct experiences of WASH-related violence or worries about WASH-related violence took place. Future studies should explore in greater detail whether experiences with WASH-related violence take place near their homes, while at school, or in other places in order to identify key sites for intervention.

The research to-date demonstrates the importance of centering the needs and concerns of women when designing and implementing water, sanitation, and hygiene interventions. Though outside the scope of this paper, the needs of additional groups that may face further marginalization/risk of violence should also be considered, such as elderly people as well as transgender and gender expansive people who likely face greater threat of violence – additional research is warranted to better understand the scope of these individuals' experiences in order to direct resources for prevention.

This study contributes to our understanding of the scope of WASH-related violence experienced by adolescent girls and young women in Mpumalanga, South Africa, and is the first quantitative study to our knowledge that documents the relationship between direct experiences of WASH-related violence and mental health. Experience of WASH-related violence is common among young women in rural South Africa, and is associated with substantially higher prevalence of depressive symptoms. Additional research to explore the experience of violence faced by women and girls when collecting water or using the toilet is needed.

For peer review only - http://bmjopen.bmj.com/site/about/guidelines.xhtml

Acknowledgements

We thank the HIV Prevention Trials Network 068 study team and all trial participants.

Authors Contribution

AP, RT, RGW, KK, JA, and SAL contributed to the overall study conceptualization, design, and implementation of data collection. RTJ, DEG, and JA conceived of the analytic plan with input from TBN, SAL, and AP. RTJ conducted the quantitative analysis. RTJ lead the writing of the manuscript, with contributions, review, and approval from all authors. All authors read and approved the final manuscript. RTJ and JA were responsible for the decision to submit the manuscript.

Competing Interests

The authors have no competing interests to declare.

Funding

This study was funded by the National Institutes of Health (grant R01 MH110186) and by the National Institute of Allergy and Infectious Diseases, the National Institute of Mental Health, and the National Institute on Drug Abuse (awards UM1 AI068619 (HPTN Leadership and Operations Center), UM1AI068617 (HIV Prevention Trials Network Statistical and Data Management Center), and UM1AI068613 (HIV Prevention Trials Network Laboratory Center)). The content is solely the responsibility of the authors and does not necessarily represent the official views of the National Institutes of Health. This work was also supported by the National Institute of Mental Health (grant R01MH087118) and the Carolina Population Center (grant P2C HD050924). Additional support was provided by the Division of Intramural Research, National Institute of Allergy and Infectious Diseases. The MRC/Wits Rural Public Health and Health Transitions Research Unit and Agincourt Health and Socio-Demographic Surveillance System, a node of the South African Population Research Infrastructure Network (SAPRIN), have been supported by the National Department of Science and Innovation, the University of the Witwatersrand, and the Medical Research Council, South Africa, and by the Wellcome Trust, United Kingdom (grants 058893/Z/99/A, 069683/Z/02/Z, 085477/Z/08/Z, and 085477/B/08/Z).

Data Sharing

No additional data available.

For peer review only

REFERENCES

1. World Health Organization. Progress on household drinking water, sanitation and hygiene 2000-2020: five years into the SDGs. 2021.
2. World Health Organization. Progress on drinking water, sanitation and hygiene: 2017 update and SDG baselines. 2017.
3. Pommells M, Schuster-Wallace C, Watt S, et al. Gender Violence as a Water, Sanitation, and Hygiene Risk: Uncovering Violence Against Women and Girls as It Pertains to Poor WaSH Access. Violence Against Women. 2018;24(15):1851-62.
4. Sommer M, Ferron S, Cavill S, et al. Violence, gender and WASH: spurring action on a complex, under-documented and sensitive topic. Environment and Urbanization. 2015;27(1):105-16.
5. Corburn J, Hildebrand C. Slum Sanitation and the Social Determinants of Women's Health in Nairobi, Kenya. Journal of Environmental and Public Health. 2015 2015/04/28;2015:209505.
6. Winter S, Barchi F, Dzombo MN. Drivers of women's sanitation practices in informal settlements in sub-Saharan Africa: a qualitative study in Mathare Valley, Kenya. International journal of environmental health research. 2018;28(6):609-25.
7. Khanna T, Das M. Why gender matters in the solution towards safe sanitation? Reflections from rural India. Glob Public Health. 2016;11(10):1185-201.
8. Kulkarni S, O'Reilly K, Bhat S. No relief: Lived experiences of inadequate sanitation access of poor urban women in India. Gender & Development. 2017;25(2):167-83.
9. Jadhav A, Weitzman A, Smith-Greenaway E. Household sanitation facilities and women's risk of non-partner sexual violence in India. BMC Public Health. 2016;16(1):016-3797.
10. Kayser GL, Chokhandre P, Rao N, et al. Household sanitation access and risk for non-marital sexual violence among a nationally representative sample of women in India, 2015-16. SSM Popul Health. 2021;13:100738-.
11. Gibbs A, Reddy T, Khanyile D, et al. Non-partner sexual violence experience and toilet type amongst young (18-24) women in South Africa: A population-based cross-sectional analysis. Glob Public Health. 2020;8:1-7.
12. Winter SC, Barchi F. Access to sanitation and violence against women: evidence from Demographic Health Survey (DHS) data in Kenya. Int J Environ Health Res. 2016;26(3):291-305.
13. Gao Y, Wang Y, Mi X, et al. Intimate Partner Violence against Women Living in Inadequate Conditions in Sub-Saharan Africa: A Meta-Analysis of Demographic and Health Survey Data. Int J Environ Res Public Health. 2021;18(19):10138.
14. Gonsalves GS, Kaplan EH, Paltiel AD. Reducing sexual violence by increasing the supply of toilets in Khayelitsha, South Africa: a mathematical model. PLoS One. 2015;10(4):e0122244.
15. Boateng GO, Collins SM, Mbullo P, et al. A novel household water insecurity scale: Procedures and psychometric analysis among postpartum women in western Kenya. PLoS One. 2018;13(6):e0198591.
16. Caruso BA, Clasen T, Yount KM, et al. Assessing Women's Negative Sanitation Experiences and Concerns: The Development of a Novel Sanitation Insecurity Measure. Int J Environ Res Public Health. 2017;14(7):755.
17. Wutich A, Brewis A, Tsai A. Water and mental health. Wiley Interdisciplinary Reviews: Water. 2020;7(5):e1461.
18. Workman CL, Ureksoy H. Water insecurity in a syndemic context: Understanding the psycho-emotional stress of water insecurity in Lesotho, Africa. Soc Sci Med. 2017;179:52-60.
19. Caruso BA, Cooper HLF, Haardörfer R, et al. The association between women's sanitation experiences and mental health: A cross-sectional study in Rural, Odisha India. SSM Popul Health. 2018;5:257-66.

For peer review only - http://bmjopen.bmj.com/site/about/guidelines.xhtml

20. Kahn K, Collinson MA, Gómez-Olivé FX, et al. Profile: Agincourt health and socio-demographic surveillance system. Int J Epidemiol. 2012;41(4):988-1001.
21. Hewett PC, Mensch BS, Erulkar AS. Consistency in the reporting of sexual behaviour by adolescent girls in Kenya: a comparison of interviewing methods. Sex Transm Infect. 2004;80(suppl 2):ii43-ii8.
22. Pettifor A, MacPhail C, Selin A, et al. HPTN 068: A Randomized Control Trial of a Conditional Cash Transfer to Reduce HIV Infection in Young Women in South Africa-Study Design and Baseline Results. AIDS Behav. 2016;20(9):1863-82.
23. Pettifor A, MacPhail C, Hughes JP, et al. The effect of a conditional cash transfer on HIV incidence in young women in rural South Africa (HPTN 068): a phase 3, randomised controlled trial. Lancet Glob Health. 2016;4(12):e978-e88.
24. Radloff LS. The CES-D scale: A self-report depression scale for research in the general population. Applied psychological measurement. 1977;1(3):385-401.
25. Vilagut G, Forero CG, Barbaglia G, et al. Screening for depression in the general population with the Center for Epidemiologic Studies Depression (CES-D): a systematic review with meta-analysis. PLoS One. 2016;11(5):e0155431.
26. Goin DE, Pearson RM, Craske MG, et al. Depression and Incident HIV in Adolescent Girls and Young Women in HIV Prevention Trials Network 068: Targets for Prevention and Mediating Factors. Am J Epidemiol. 2019;189(5):422-32.
27. Brewis A, Workman C, Wutich A, et al. Household water insecurity is strongly associated with food insecurity: evidence from 27 sites in low-and middle-income countries. American Journal of Human Biology. 2020;32(1):e23309.
28. LP SC. Stata Statistical Software Release 15: Stata Press Publication; 2017.
29. MRC/Wits-Agincourt Unit. Public Engagement. [May 6, 2022]; Available from: https://www.agincourt.co.za/?page_id=1913.
30. Barchi F, Winter SC. Non-Partner Violence in Sub-Saharan Africa and the Built Environment: A Multicountry Analysis of the Effects of Sanitation, Water Access, and Urban Settings. Violence Against Women. 2020;26(10):1101-19.
31. Abrahams N, Devries K, Watts C, et al. Worldwide prevalence of non-partner sexual violence: a systematic review. Lancet. 2014;383(9929):1648-54.
32. Beydoun HA, Beydoun MA, Kaufman JS, et al. Intimate partner violence against adult women and its association with major depressive disorder, depressive symptoms and postpartum depression: a systematic review and meta-analysis. Soc Sci Med. 2012;75(6):959-75.
33. Domenech Del Rio I, Sirvent Garcia Del Valle E. Non-partner sexual violence against women in Spain: lifetime prevalence, perpetrators and consequences on mental health. J Public Health. 2017;39(4):738-44.
34. Hankin CS, Skinner KM, Sullivan LM, et al. Prevalence of depressive and alcohol abuse symptoms among women VA outpatients who report experiencing sexual assault while in the military. J Trauma Stress. 1999;12(4):601-12.
35. Kimerling R, Gima K, Smith MW, et al. The Veterans Health Administration and military sexual trauma. Am J Public Health. 2007;97(12):2160-6.
36. Jordan CE, Campbell R, Follingstad D. Violence and women's mental health: the impact of physical, sexual, and psychological aggression. Annual review of clinical psychology. 2010;6.
37. Shiras T, Cumming O, Brown J, et al. Shared latrines in Maputo, Mozambique: exploring emotional well-being and psychosocial stress. BMC Int Health Hum Rights. 2018;18(1):018-0169.
38. Gilman SE, Trinh NH, Smoller JW, et al. Psychosocial stressors and the prognosis of major depression: a test of Axis IV. Psychol Med. 2013;43(2):303-16.

For peer review only - http://bmjopen.bmj.com/site/about/guidelines.xhtml

39. Corburn J, Karanja I. Informal settlements and a relational view of health in Nairobi, Kenya: Sanitation, gender and dignity. Health promotion international. 2014;31(2):258-69.
40. Sahoo KC, Hulland KR, Caruso BA, et al. Sanitation-related psychosocial stress: A grounded theory study of women across the life-course in Odisha, India. Soc Sci Med. 2015;139:80-9.
41. Joshi D, Fawcett B, Mannan F. Health, hygiene and appropriate sanitation: experiences and perceptions of the urban poor. Environment and Urbanization. 2011;23(1):91-111.
42. Pretorius TB. Cross-cultural application of the Center for Epidemiological Studies Depression Scale: a study of black South African students. Psychol Rep. 1991;69(3 Pt 2):1179-85.
43. Kilburn K, Prencipe L, Hjelm L, et al. Examination of performance of the Center for Epidemiologic Studies Depression Scale Short Form 10 among African youth in poor, rural households. BMC psychiatry. 2018;18(1):1-13.

For peer review only

STROBE Statement—Checklist of items that should be included in reports of ***cohort studies***

	Item No	Recommendation	Page No
**Title and abstract**	1	(*a*) Indicate the study's design with a commonly used term in the title or the abstract	1
		(*b*) Provide in the abstract an informative and balanced summary of what was done and what was found	2
**Introduction**			
Background/rationale	2	Explain the scientific background and rationale for the investigation being reported	3
Objectives	3	State specific objectives, including any prespecified hypotheses	3-4
**Methods**			
Study design	4	Present key elements of study design early in the paper	4
Setting	5	Describe the setting, locations, and relevant dates, including periods of recruitment, exposure, follow-up, and data collection	4-5
Participants	6	(*a*) Give the eligibility criteria, and the sources and methods of selection of participants. Describe methods of follow-up	4-5
		(*b*) For matched studies, give matching criteria and number of exposed and unexposed	
Variables	7	Clearly define all outcomes, exposures, predictors, potential confounders, and effect modifiers. Give diagnostic criteria, if applicable	5
Data sources/ measurement	8*	For each variable of interest, give sources of data and details of methods of assessment (measurement). Describe comparability of assessment methods if there is more than one group	5
Bias	9	Describe any efforts to address potential sources of bias	5
Study size	10	Explain how the study size was arrived at	4
Quantitative variables	11	Explain how quantitative variables were handled in the analyses. If applicable, describe which groupings were chosen and why	5
Statistical methods	12	(*a*) Describe all statistical methods, including those used to control for confounding	5
		(*b*) Describe any methods used to examine subgroups and interactions	n/a
		(*c*) Explain how missing data were addressed	5
		(*d*) If applicable, explain how loss to follow-up was addressed	5
		(*e*) Describe any sensitivity analyses	n/a
**Results**			
Participants	13*	(a) Report numbers of individuals at each stage of study—eg numbers potentially eligible, examined for eligibility, confirmed eligible, included in the study, completing follow-up, and analysed	6
		(b) Give reasons for non-participation at each stage	6
		(c) Consider use of a flow diagram	n/a
Descriptive data	14*	(a) Give characteristics of study participants (eg demographic, clinical, social) and information on exposures and potential confounders	6-7
		(b) Indicate number of participants with missing data for each variable of interest	6
		(c) Summarise follow-up time (eg, average and total amount)	
Outcome data	15*	Report numbers of outcome events or summary measures over time	6-7

For peer review only

For peer review only - http://bmjopen.bmj.com/site/about/guidelines.xhtml

Main results	16	(*a*) Give unadjusted estimates and, if applicable, confounder-adjusted estimates and their precision (eg, 95% confidence interval). Make clear which confounders were adjusted for and why they were included	6-8
		(*b*) Report category boundaries when continuous variables were categorized	n/a
		(*c*) If relevant, consider translating estimates of relative risk into absolute risk for a meaningful time period	8
Other analyses	17	Report other analyses done—eg analyses of subgroups and interactions, and sensitivity analyses	
**Discussion**			
Key results	18	Summarise key results with reference to study objectives	8
Limitations	19	Discuss limitations of the study, taking into account sources of potential bias or imprecision. Discuss both direction and magnitude of any potential bias	9-10
Interpretation	20	Give a cautious overall interpretation of results considering objectives, limitations, multiplicity of analyses, results from similar studies, and other relevant evidence	10
Generalisability	21	Discuss the generalisability (external validity) of the study results	10
**Other information**			
Funding	22	Give the source of funding and the role of the funders for the present study and, if applicable, for the original study on which the present article is based	11

*Give information separately for exposed and unexposed groups.

Note: An Explanation and Elaboration article discusses each checklist item and gives methodological background and published examples of transparent reporting. The STROBE checklist is best used in conjunction with this article (freely available on the Web sites of PLoS Medicine at http://www.plosmedicine.org/, Annals of Internal Medicine at http://www.annals.org/, and Epidemiology at http://www.epidem.com/). Information on the STROBE Initiative is available at http://www.strobe-statement.org.

For peer review only
